# Elevated Circulating Pigment Epithelium-Derived Factor Predicts the Progression of Diabetic Nephropathy in Patients With Type 2 Diabetes

**DOI:** 10.1210/jc.2014-2235

**Published:** 2014-08-28

**Authors:** Elaine Hui, Chun-Yip Yeung, Paul C.H. Lee, Yu-Cho Woo, Carol H.Y. Fong, Wing-Sun Chow, Aimin Xu, Karen S.L. Lam

**Affiliations:** Department of Medicine (E.H., C.-Y.Y., P.C.H.L., Y.-C.W., C.H.Y.F., W.-S.C., A.X., K.S.L.L., Queen Mary Hospital; Research Centre of Heart, Brain, Hormones, and Healthy Aging (E.H., A.X., K.S.L.L.); and State Key Laboratory of Pharmaceutical Biotechnology (A.X., K.S.L.L.), The University of Hong Kong, Hong Kong, China

## Abstract

**Context:**

Pigment epithelium-derived factor (PEDF), a circulating glycoprotein with antiangiogenic, antioxidative, and anti-inflammatory properties, protects against diabetic nephropathy (DN) in animal models.

**Objective:**

We investigated whether circulating PEDF predicted the progression of DN in a 4-year prospective study.

**Design, Setting, and Participants:**

Baseline plasma PEDF levels were measured in type 2 diabetic subjects recruited from the Hong Kong West Diabetes Registry. The role of PEDF in predicting chronic kidney disease (CKD) and albuminuria progression was analyzed using Cox regression analysis.

**Main Outcome Measure:**

We evaluated CKD progression, defined as deterioration in CKD staging and a 25% or greater drop in estimated glomerular filtration rate (eGFR) according to International Society of Nephrology statements.

**Results:**

At baseline, plasma PEDF levels increased progressively with CKD staging (*P* for trend <.001; n = 1136). Among 1071 subjects with baseline CKD stage ≤3, plasma PEDF levels were significantly higher in those with CKD progression (n = 171) during follow-up than those without (*P* < .001). Baseline PEDF was independently associated with CKD progression (hazard ratio = 2.76; 95% confidence interval = 1.39–5.47; *P* = .004), adjusted for age, sex, waist circumference, diabetes duration, hemoglobin A1c, systolic blood pressure, use of antihypertensive drugs, C-reactive protein, and eGFR. Elevated baseline PEDF was also associated with the development of microalbuminuria/albuminuria in a subgroup with normoalbuminuria and eGFR >60 mL/min/1.73 m^2^ (n = 462) at baseline (hazard ratio = 2.75; 95% confidence interval = 1.01–7.49; *P* < .05), even after adjustment for potential confounders.

**Conclusions:**

Elevated PEDF levels may represent a compensatory change in type 2 diabetic patients with renal disease and appear to be a useful marker for evaluating the progression of DN.

Pigment epithelium-derived factor (PEDF), a member of the serine protease inhibitor (serpin) gene family, is a 50-kDa secreted glycoprotein first identified in the conditioned medium of human retinal pigment epithelial cells as a neurotrophic factor and a potent angiogenic inhibitor (1). Cell-based and animal studies have suggested PEDF to be a local protective factor against diabetic microvascular damage (2, 3). Decreased PEDF protein and mRNA expression has been found in kidneys of diabetic mice (3). PEDF has also been shown to suppress the expression of fibrogenic (4), proinflammatory, and angiogenic factors (5, 6), thus contributing to pathological changes in early diabetic nephropathy (DN).

Identification of novel biomarkers implicated in DN may enable early detection of patients at risk of clinical disease progression, especially before a significant reduction in glomerular filtration rate (GFR) or the development of microalbuminuria. In the current prospective study, our goal was to examine the role of plasma PEDF as a biomarker for the detection of chronic kidney disease (CKD) progression in patients with type 2 diabetes mellitus (T2DM) before progressing to severely reduced kidney function. In a subset of patients with a normal urine albumin-to-creatinine ratio, we also assessed whether baseline plasma PEDF levels can predict the development of microalbuminuria/albuminuria.

## Subjects and Methods

### Subjects

Subjects were recruited from the Hong Kong West Diabetes Registry between 2008 and 2013. All unrelated subjects with T2DM who attended the Diabetes Clinic at the Queen Mary Hospital were recruited consecutively to participate in a prospective study to identify the risk factors predisposing to the development of diabetic complications. Each visit comprised clinical assessments and laboratory investigations to determine the control of diabetes and related cardiovascular risk factors, and the presence of diabetic complications. Inclusion criteria were T2DM, age ≥30 years, Chinese nationality, and being able to give informed consent. Diabetes was defined according to the 2008 American Diabetes Association diagnostic criteria (7). Patients were excluded if they were on dialysis or had received a kidney transplant at baseline. A total of 1136 T2DM subjects, who attended regular visits at least twice a year, with the latest follow-up in or before October 2013, were enrolled in the study. Part 1 was to examine the cross-sectional relationships between baseline PEDF and other variables at baseline, including CKD staging. In part 2A, to examine prospectively the role of baseline PEDF in the early identification of diabetic patients at increased risk of CKD progression, only subjects with CKD stage 1 to 3 were included and those with established severely reduced kidney function, ie, CKD stage 4 and 5, were excluded. In part 2B, to examine prospectively the relationship between baseline PEDF and the development of microalbuminuria/albuminuria, only subjects with normoalbuminuria and estimated GFR (eGFR) >60 mL/min/1.73 m^2^ at baseline were included.

### Endpoint definitions

The primary endpoint was CKD progression based on the International Society of Nephrology recommendation statements (8). CKD progression was defined as a decline in GFR category (≥90 [stage 1], 60–89 [stage 2], 45–59 [stage 3a], 30–44 [stage 3b], 15–29 [stage 4], or <15 [stage 5] mL/min/1.73 m^2^), accompanied by a 25% or greater deterioration in eGFR from baseline (8). eGFR was calculated using the Modification of Diet in Renal Disease (MDRD) Study formula and expressed in milliliters per minute per 1.73 m^2^: eGFR = 175 × [serum creatinine (in μmol/L) × 0.011]^−1.154^ × [age]^−0.203^ (× 0.742 if female). The isotope dilution mass spectrometry-traceable version of the MDRD Study equation was used (9). According to the 2013 Kidney Disease: Improving Global Outcomes guidelines (10), the estimation of GFR was considered to be more accurate using the Chronic Kidney Disease Epidemiology Collaboration (CKD-EPI) equation compared with the MDRD formula, particularly for values >60 mL/min/1.73 m^2^. Furthermore, because the CKD-EPI equation has been externally validated in Chinese CKD patients (11), we also calculated eGFR using the CKD-EPI equation. The CKD-EPI equation was expressed as GFR = 141 × min(SCr/κ,1)^α^ × max(SCr/κ,1)^−1.209 ×^ 0.993^age^ × 1.018 (if female), where SCr is serum creatinine, κ is 0.7 for females and 0.9 for male, α is −0.329 for females and −0.411 for males, min indicates the minimum of SCr/κ or 1, and max indicates the maximum of SCr/κ or 1 (12). The secondary endpoint was the progression from normoalbuminuria to microalbuminuria/albuminuria, as defined by a urine albumin-to-creatinine ratio of 30 μg/mg creatinine (3.5 mg/mmol creatinine) or above on spot urine analysis in at least 2 samples on 2 separate occasions within 6 months (13).

### Clinical and biochemical assessments

Subjects attended each visit after an overnight fast of at least 8 hours. At the baseline visit, demographic data, including age, sex, occupation, smoking, alcohol consumption, and physical activity were ascertained. Detailed medical, medication, and family histories were obtained using a standardized questionnaire. Duration of diabetes was calculated from the time of diagnosis to the time of study visit at baseline. Anthropometric parameters, including body weight, height, body mass index (BMI), waist circumference (WC), and blood pressure (BP) were measured. Blood was drawn for fasting plasma glucose, lipids, serum creatinine, hemoglobin A1c (HbA1c), and C-reactive protein (CRP). PEDF was measured with an ELISA (BioVendor Laboratory Medicine, Inc), with calibration and quality control performed as previously reported (14). Intra- and interassay coefficients of variation for this assay were 2.9% to 4.1% and 5.3% to 6.6%, respectively.

All subjects gave informed consent, and the present study was approved by the Ethics Committee of the Faculty of Medicine, University of Hong Kong.

### Statistical analysis

All statistical analyses were performed using SPSS Statistics version 19. Data that were not normally distributed, as determined using the Kolmogorov-Smirnov test, were natural-logarithmically transformed to obtain near normality before analysis. Values are reported as means ± SD or medians with interquartile range (IQR) as appropriate. The χ^2^ test and one-way ANOVA were used for comparisons of categorical and continuous variables, respectively. Correlation between plasma PEDF and clinical/biochemical variables was examined by Pearson or partial correlation analysis. Multivariable ordinal regression analysis was used to identify the factors with independent association with CKD staging at baseline. Kaplan-Meier curves of CKD progression were generated for subjects by quartiles of PEDF concentration and analyzed by the log-rank test. Multivariable Cox regression analysis was used to estimate the hazard ratios (HRs) and 95% confidence intervals (CIs) for CKD progression and progression in urine albumin excretion (UAE). The variables included in the multivariate regression models were those that were statistically significant in univariate analyses after correcting for multiple comparisons by using Bonferroni correction, or were biologically relevant. In all statistical tests, two-sided *P* values <.05 were considered significant.

## Results

### Part 1

**Figure 1.**
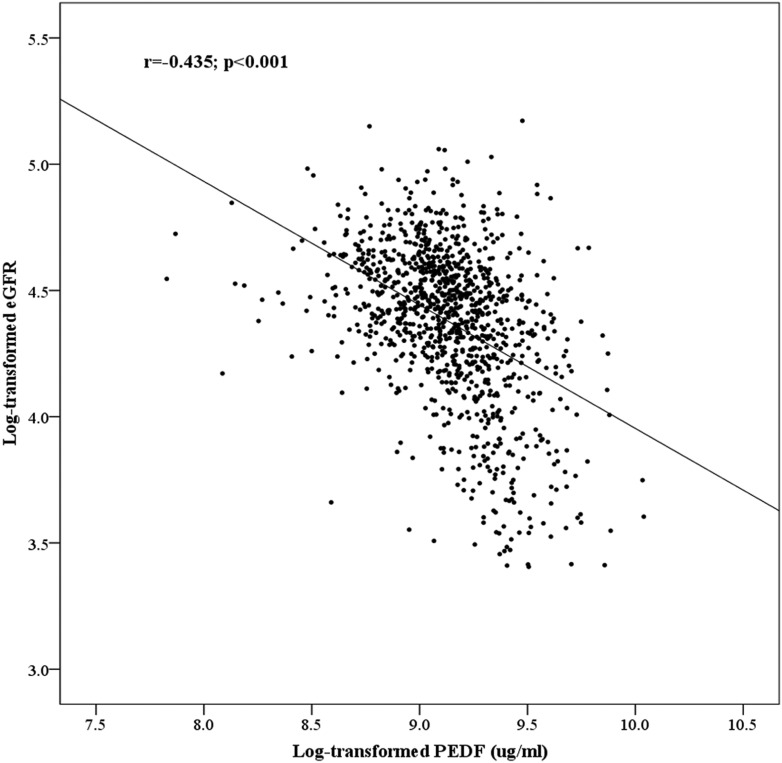
Correlation between PEDF and eGFR at baseline.

Among the 1136 T2DM subjects, plasma PEDF level correlated significantly with eGFR (*r* = −0.435, *P* < .001) (Figure 1) and increased progressively with increasing CKD staging at baseline (*P* for trend < .001) (Supplemental Table 1). It also increased with the presence of microalbuminuria/albuminuria at baseline (*P* < .001 vs normoalbuminuria) and, in accordance with our previous study (15), with age (*r* = 0.09, *P* = .003), and was higher in men (median 9.58 [IQR 8.22–11.32] vs 9.19 [7.59–10.93] μg/mL in women; *P* = .012). Furthermore, plasma PEDF correlated significantly with other metabolic parameters including BP, DM duration, HbA1c, triglycerides, high-density lipoprotein cholesterol, BMI, and WC (all *P* < .05) as well as with hypertension/use of antihypertensive and use of lipid-lowering drugs (all *P* < .001) on univariate analysis (data not shown). These metabolic parameters, apart from BMI and WC, also varied significantly with CKD staging (Supplemental Table 1). As previously reported (16, 17), the plasma PEDF level was higher in the presence of cardiovascular diseases (CVD) (known coronary artery disease or stroke) at baseline (*P* < .001 vs no CVD). However, on multivariable ordinal regression analysis, CVD was not significantly associated with CKD staging, whereas PEDF remained significantly associated with CKD stages (adjusted *P* < .001), together with age (*P* < .001) and microalbuminuria/albuminuria (*P* = .014), suggesting the association of PEDF with CKD staging to be independent of that between PEDF and CVD.

### Part 2A

**Figure 2.**
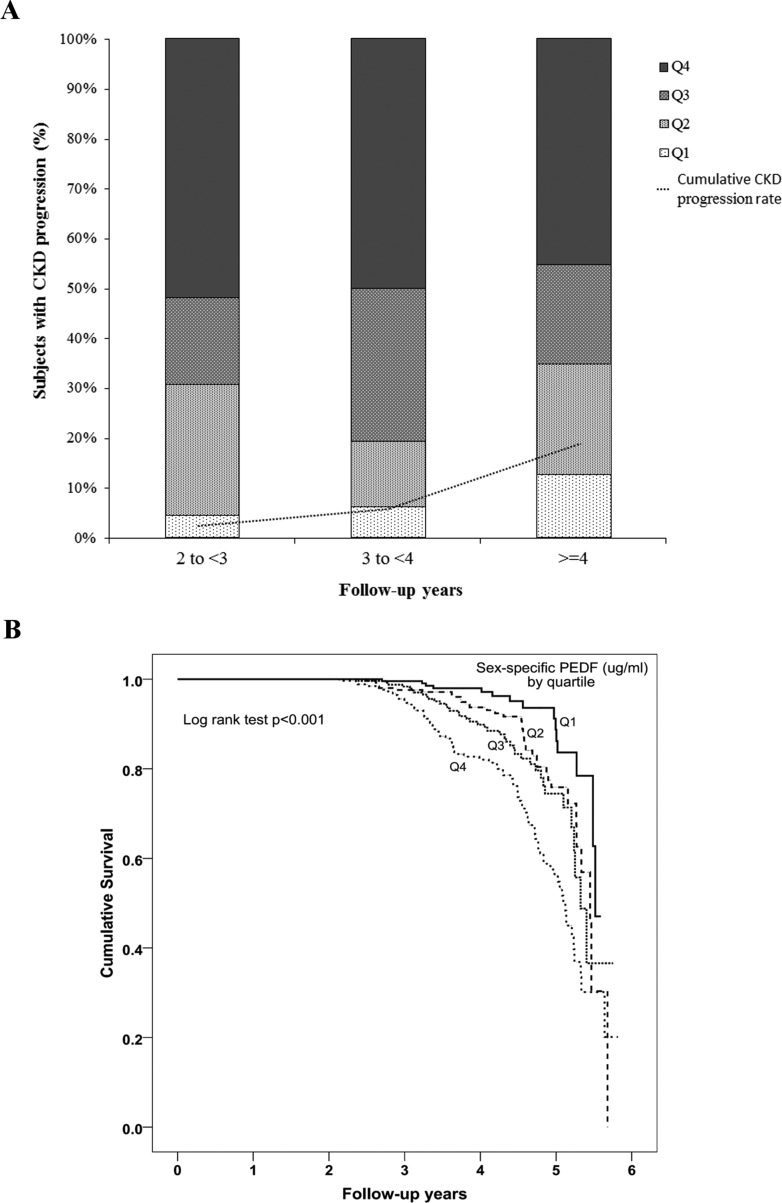
A, Percentage of subjects showing CKD progression at each assessment according to their respective sex-specific PEDF quartile. The dotted line indicates the cumulative CKD progression rate. B, Cumulative survival curves (Kaplan-Meier estimates) showing CKD progression over a median follow-up of 4 years in relation to baseline PEDF concentration in sex-specific quartiles.

The relationship between baseline plasma PEDF and nephropathy progression was examined in 1071 subjects, after excluding 39 subjects with less than 2 years of follow-up and 26 subjects with CKD stage 4 or 5. After a median interval of 4.0 years (IQR 3.2–4.7), among the 1071 subjects with baseline CKD stage ≤3, 171 (16.0%; Figure 2A) had CKD progression. As shown in Table 1, the CKD progressors were older, had higher systolic BP (SBP), higher prevalence rates of hypertension (92.4% vs 72.1%), more use of antihypertensive drugs, and higher triglycerides and CRP (all *P* < .001, vs subjects with no CKD progression). They had a longer duration of diabetes (*P* < 0.001) and higher baseline HbA1c (*P* = .008). They also had significantly higher baseline serum creatinine, lower eGFR, and higher prevalence of microalbuminuria/albuminuria (66.5% vs. 27.8%) (all *P* < 0.001). Baseline plasma PEDF levels were significantly higher in subjects with CKD progression than those without (*P* < .001, Table 1). Figure 2A shows the percentage of subjects with CKD progression at each assessment according to their respective PEDF quartile. The risk of CKD progression increased progressively across the sex-specific quartiles of baseline plasma PEDF level (*P* < .001, log-rank test), as shown in Figure 2B.

**Table 1.** Baseline Characteristics of the 1071 Participants by CKD Progression

Baseline Parameters	CKD Progression		*P* Value
	Yes	No	
n	171	900	
Sex (male, %)	52.6	58.3	.171
Age, y	57.2 ± 8.66	54.5 ± 9.32	<.001
BMI, kg/m^2^	26.2 ± 4.64	25.8 ± 4.52	.242
WC, cm			.013^c^
M	93.0 ± 11.1	90.9 ± 10.8	
F	89.4 ± 12.7	86.6 ± 12.4	
Ever smoke, %	37.4	32.4	.205
SBP, mm Hg	139.1 ± 20.3	130.1 ± 19.2	<.001
DBP, mm Hg	77.7 ± 10.5	78.5 ± 9.57	.328
HT, %	92.4	72.1	<.001
Anti-hypertensive drug, %	90.6	68.1	<.001
ACEI/ARB, %	78.9	56.4	<.001
DM duration, y	15.3 ± 8.53	12.5 ± 7.75	<.001
FPG (mmol/liter)	8.53 ± 2.65	8.34 ± 2.68	.381
HbA1c, %	8.63 ± 1.73	8.28 ± 1.56	.008
Total cholesterol, mmol/L	4.93 ± 1.19	4.72 ± 0.95	.011
TG, mmol/L^b^	1.57 (1.19–2.10)	1.28 (0.91–1.90)	<.001
HDL-C, mmol/L	1.19 ± 0.38	1.20 ± 0.34	.907
LDL-C, mmol/L	2.90 ± 0.95	2.80 ± 0.83	.146
Lipid-lowering drug, %	33.3	30.6	.472
Serum creatinine, μmol/L^b^			<.001^c^
Males	96.0 (78.5–127.3)	82.0 (74.0–96.0)	
Females	69.0 (56.0–79.0)	62.0 (55.0–73.0)	
eGFR^b^	72.6 (56.5–93.3)	85.1 (70.7–98.3)	<.001
CKD stage			<.001
1	26.3	38.7	
2	44.4	48.1	
3a	14.0	8.6	
3b	15.2	4.7	
DN,^d^ %			<.001
Normal	33.5	72.2	
Micro	29.7	21.8	
Clinical albuminuria	36.8	6.0	
Micro/clinical albuminuria,^d^ %	66.5	27.8	<.001
PEDF,^b^ μg/mL			<.001^c^
Males	10.70 (9.12–12.59)	9.38 (8.00–11.00)	
Females	10.48 (8.89–12.86)	8.75 (7.39–10.36)	
CRP,^b^ mg/L	1.84 (0.92–4.31)	1.34 (0.50–2.96)	<.001

Abbreviations: ACEI, angiotensin-converting enzyme inhibitor; ARB, angiotensin receptor blocker; DBP, diastolic BP; FPG, fasting plasma glucose; HDL-C, high-density lipoprotein cholesterol; LDL-C, low-density lipoprotein cholesterol; TG, triglycerides.

a Data are presented as mean ± SD or median (IQR).

b Log-transformed before analysis.

c Sex-adjusted *P* value.

d Data for n = 928 were available for analysis.

The independent predictors for CKD progression were identified using multivariable Cox regression analysis. As shown in Table 2, baseline PEDF concentration was an independent predictor of CKD progression (adjusted HR = 2.76; 95% CI = 1.39–5.47; *P* = .004), together with SBP (HR = 1.03; 95% CI = 1.02–1.03; *P* < .001), duration of diabetes (HR = 1.04; 95% CI = 1.02–1.06; *P* < .001), CRP (HR = 1.18; 95% CI = 1.03–1.36; *P* = .020), and the use of antihypertensive drug(s) (HR = 2.31; 95% CI = 1.31–4.07; *P* = .004), in a model that also included age, sex, WC, HbA1c, and eGFR (Table 2). Baseline PEDF remained an independent predictor of CKD progression (adjusted HR = 2.75; 95% CI = 1.39–5.46; *P* = .004), in a model that included serum creatinine in place of eGFR (data not shown). Analyses were repeated with the use of antihypertensive treatment being replaced by the use of angiotensin-converting enzyme inhibitor or angiotensin receptor blocker, PEDF remained significant in the Cox regression models (adjusted HR = 2.77; 95% CI = 1.39–5.51; *P* = .004).

**Table 2.** Multivariable Cox Regression Analysis Showing the Association of the PEDF With CKD Progression^a^

Baseline Variables	Adjusted HR (95% CI)	*P* Value
Sex (male)	0.82 (0.60–1.12)	.218
Age, y	1.01 (0.98–1.03)	.601
WC, cm	1.01 (0.99–1.02)	.291
DM duration, y	1.04 (1.02–1.06)	<.001
HbA1c, %	0.95 (0.86–1.04)	.257
SBP, mm Hg	1.03 (1.02–1.03)	<.001
Antihypertensive treatment, %	2.31 (1.31–4.07)	.004
eGFR^b^	0.76 (0.44–1.30)	.318
CRP,^b^ mg/L	1.18 (1.03–1.36)	.020
PEDF,^b^ μg/mL	2.76 (1.39–5.47)	.004

a Multivariable Cox regression model included age, SBP, antihypertensive treatment, diabetes (DM) duration, eGFR, CRP and PEDF (significant variables after Bonferroni correction in the univariate analyses), together with sex, WC and HbA1c (biological relevant variables). PEDF remained significant if the use of anti-hypertensive treatment was replaced by the use of angiotensin-converting enzyme inhibitor or angiotensin receptor blocker (*P* = .004).

b Log-transformed before analysis.

When CKD-EPI equation was used to estimate GFR, 1073 subjects were classified as CKD stage 1 to 3, of which 166 had CKD progression. Multivariable Cox regression analysis showed that PEDF independently predicted CKD progression (adjusted HR = 3.77; 95% CI = 1.79–7.95; *P* < .001) in a model that included sex, age, WC, diabetes duration, HbA1c, SBP, antihypertensive treatment, CRP, and eGFR using CKD-EPI (eGFR-EPI). When eGFR-EPI was substituted with baseline CKD stage, creatinine, or the presence of baseline microalbuminuria/albuminuria, the association of PEDF with CKD progression remained significant (adjusted HR = 3.70; 95% CI = 1.77–7.74; and adjusted HR = 5.11; 95% CI = 2.42–10.8; both *P* < .001 for creatinine and CKD stage, respectively; adjusted HR = 2.34; 95% CI = 1.15–4.77; *P* = .019 for baseline microalbuminuria/albuminuria).

#### Part 2B

Elevated PEDF was also associated with the development of microalbuminuria/albuminuria in a subgroup of 462 subjects with normoalbuminuria and eGFR >60 mL/min/1.73 m^2^ at baseline. Of these, 94 (20.3%) had progressed to microalbuminuria/albuminuria after a median follow-up of 4 years. Baseline PEDF levels were significantly higher in subjects with progression in UAE than those without (*P* < .001) (Table 3). Using multivariable Cox regression analysis, including baseline variables that were significantly different or biologically relevant between subjects with progression in UAE and those without, baseline plasma PEDF levels were associated with UAE progression (adjusted HR = 2.75; 95% CI = 1.01–7.49; *P* < .05), together with SBP (HR = 1.02; 95% CI = 1.01–1.03; *P* = .003) (Table 4). Using eGFR-EPI, 98 of 474 subjects progressed to microalbuminuria/albuminuria. PEDF was marginally associated with microalbuminuria/albuminuria progression (adjusted HR = 2.63; 95% CI = 0.98–7.08; *P* = .055), adjusted for sex, age, WC, diabetes duration, HbA1c, SBP, antihypertensive treatment, CRP, and eGFR-EPI. When baseline serum creatinine or CKD stage was substituted for eGFR-EPI, elevated PEDF level remained a significant predictor of microalbuminuria/albuminuria progression (adjusted HR = 2.68; 95% CI = 1.00–7.20; *P* = .05; and HR = 3.19; 95% CI = 1.19–8.53; *P* = .021 for creatinine and CKD stage, respectively).

**Table 3.** Baseline Characteristics of the 462 Participants With Microalbuminuria/Albuminuria Progression

	Progressors	Nonprogressors	*P* Value
n	94	368	
Sex (male, %)	42.6	53.5	.057
Age, y	55.8 ± 9.09	53.3 ± 8.88	.014
Ever smoke, %	27.7	26.9	.883
BMI, kg/m^2^	25.5 ± 4.32	25.0 ± 4.03	.245
WC (cm)			.095^b^
Males	90.4 ± 13.6	88.4 ± 9.76	
Females	86.5 ± 10.5	84.3 ± 11.8	
SBP, mm Hg	132.4 ± 17.9	126.4 ± 17.8	.004
DBP, mm Hg	77.6 ± 8.60	76.9 ± 8.62	.509
Hypertension, %	78.7	59.2	<.001
Antihypertensive treatment, %	75.5	54.3	<.001
ACEI/ARB, %	68.1	41.8	<.001
DM duration, y	13.7 ± 7.46	12.3 ± 7.76	.123
FPG, mmol/L	8.39 ± 2.74	8.14 ± 2.44	.388
HbA1c, %	8.61 ± 1.50	8.02 ± 1.34	<.001
Total cholesterol, mmol/L	4.82 ± 1.00	4.78 ± 0.99	.720
TG,^a^ mmol/L	1.42 (1.02–1.81)	1.17 (0.84–1.91)	.219
HDL-C, mmol/L	1.23 ± 0.34	1.24 ± 0.36	.710
LDL-C, mmol/L	2.89 ± 0.93	2.84 ± 0.84	.606
Lipid-lowering treatment, %	28.7	23.1	.256
Creatinine,^a^ μmol/L			.023^b^
Males	81.0 (72.5–91.5)	79.0 (72.0–87.5)	
Females	63.0 (56.0–72.3)	89.9 (79.9–102.0)	
eGFR^a^	85.0 (73.5–98.0)	89.5 (79.2–100.6)	.010
CKD stage, %			.199
1	40.4	47.8	
2	59.6	52.2	
PEDF,^a^ μg/mL			<.001^b^
M	9.25 (8.35–10.45)	8.68 (7.40–9.60)	
F	8.85 (8.06–9.94)	8.22 (6.91–9.73)	
CRP,^a^ mg/L	1.27 (0.70–3.84)	1.03 (0.42–2.48)	.010

Abbreviations: ACEI, angiotensin-converting enzyme inhibitor; ARB, angiotensin receptor blocker; DBP, diastolic BP; FPG, fasting plasma glucose; HDL-C, high-density lipoprotein cholesterol; LDL-C, low-density lipoprotein cholesterol; TG, triglycerides.

a Log-transformed before analysis.

b Sex-adjusted *P* value.

**Table 4.** Multivariable Cox Regression Analysis Showing the Association of the PEDF With Microalbuminuria/Albuminuria Progression^a^

Baseline Variables	Adjusted HR (95% CI)	*P* Value
Sex (male)	0.72 (0.47–1.10)	0.129
Age, y	1.01 (0.98–1.04)	0.595
WC, cm	1.01 (0.99–1.03)	0.326
DM duration, y	0.99 (0.96–1.03)	0.702
HbA1c, %	1.10 (0.95–1.27)	0.193
SBP, mm Hg	1.02 (1.01–1.03)	0.003
Antihypertensive treatment, %	1.40 (0.81–2.40)	0.225
eGFR^b^	0.37 (1.00–1.37)	0.137
PEDF,^b^ μg/mL	2.75 (1.01–7.49)	0.048

a Multivariable Cox regression model included HbA1c, antihypertensive treatment, and PEDF (significant variables after Bonferroni correction in the univariate analyses), together with sex, age, WC, DM duration, SBP, and eGFR (biologically relevant variables).

b Log-transformed before analysis.

## Discussion

In this longitudinal observational study, we have shown for the first time that high baseline plasma PEDF levels predicted CKD progression in a large cohort of T2DM without advanced nephropathy. Moreover, baseline plasma PEDF was independently associated with the progression to microalbuminuria/albuminuria in patients with normoalbuminuria and eGFR >60 mL/min/1.73 m^2^. These findings would suggest a pathogenic link between PEDF and the development of DN in humans.

Plasma PEDF concentration was shown to be closely associated with CKD staging at baseline in the current study. This is consistent with other cross-sectional studies where circulating PEDF levels were positively correlated with creatinine (18) and negatively correlated with eGFR in both general (19) and type 2 diabetic populations (20). Serum PEDF has also been shown to be independently correlated with albumin excretion rate in 132 T2DM patients, with higher serum PEDF related to the severity of proteinuria (21). Similarly, plasma PEDF levels were significantly higher in type 2 diabetic patients with proliferative diabetic retinopathy than in nondiabetic controls (22). Our data are in line with the findings of the above cross-sectional studies and suggests that elevated circulating PEDF levels may represent a counterregulatory response to the presence of renal injury in DN.

Animal models have demonstrated that PEDF inhibits advanced glycation end-products (AGE)-induced mesangial and proximal renal tubular cell damage by blocking the gene expression of receptor for AGE (RAGE), leading to reduced reactive oxygen species, monocyte chemoattractant protein-1, TGF-β, fibronectin, and type IV collagen mRNA levels in proximal tubular cells (23, 24). PEDF also blocks apoptotic cell death of AGE-exposed podocytes essential for maintaining the structural homeostasis in the glomeruli (25). Podocyte injury results in glomerular hyperfiltration and increased albumin excretion rate and ultimately leads to glomerular sclerosis seen in DN (26). In humans, circulating AGE levels independently correlated with serum PEDF levels in 196 subjects from a general population (18). Taken together, our findings suggest that plasma PEDF may reflect a compensatory response to glomerular injury in patients with T2DM and thereby predicts the progression in UAE and subsequent deterioration in GFR.

On the other hand, PEDF was well-recognized to be associated with insulin resistance and diabetes. Clinical studies reported that circulating PEDF levels were significantly higher in subjects with T2DM than in nondiabetic controls (22, 27). The predominant sources of circulating PEDF are thought to be the adipose tissue and liver (28). We previously found that plasma PEDF concentration was independently associated with obesity-related metabolic syndrome and hypertension (15, 29). Others also reported that plasma PEDF was positively associated with obesity indices and diabetic vascular complications (22, 30). However, the pathophysiological role of PEDF in insulin resistance is not entirely clear. Some studies have shown PEDF to inhibit AGE-induced hepatic insulin resistance and restore the reduced adiponectin levels in AGE-exposed adipocytes (31, 32), whereas others have suggested that PEDF could induce insulin resistance in muscle and fat cells (28, 33). Therefore, as an alternative explanation, it is possible that elevated plasma PEDF may have contributed to worsening insulin resistance and diabetic control, which may then lead to progression in nephropathy. Whether plasma PEDF plays a compensatory or enhancing role in the pathogenesis of DN in humans is still inconclusive. Nonetheless, the current study demonstrated that PEDF remained an independent predictor of DN progression, even after adjusting for HbA1c and duration of diabetes, as well as WC, a factor related to visceral obesity and insulin resistance.

Although high PEDF levels may also reflect impaired renal metabolism, the clearance of PEDF in humans has not yet been fully characterized. PEDF is a glycoprotein with a molecular mass of 50 kDa, similar to that of albumin (65 kDa) (1). PEDF may undergo glomerular filtration and tubular reabsorption similar to that of albumin. Nevertheless, Chen et al (34) has demonstrated that urinary PEDF was independently associated with PEDF expression in the rat kidney. The same study also showed that urinary PEDF levels, which correlated with serum PEDF, were higher in diabetic patients with microalbuminuria, suggesting that an increase in PEDF expression in the kidneys of DN patients may account for the raised urinary and serum PEDF levels (34). Therefore, elevated serum PEDF does not appear to be fully dependent on impaired renal metabolism or excretion. Whether local PEDF production in the kidney is responsible for the elevated circulating levels in humans needs to be further explored. Further studies examining renal tissue biopsies would be useful to ascertain whether elevated plasma PEDF correlates with renal PEDF overexpression and production.

The use of the current definition of CKD progression has been validated using data from the Alberta Kidney Disease Network, as a decline in GFR category combined with a 25% or greater drop in GFR from baseline measurement during a median follow-up of 2.4 years (35). This is clinically meaningful because those with such progression were associated with a 2-fold increase in the risk of all-cause mortality and a 5-fold increase in the risk of end-stage renal failure (35, 36). The present study showed that plasma PEDF was independently associated with CKD progression in the multivariate analysis. As such, plasma PEDF could be considered a potential clinical marker for identifying T2DM patients who are likely to experience a decline in renal function, before they develop stage 4 or 5 CKD. Recently, in a longitudinal study on 246 male Veterans Affairs Diabetes Trial subjects, serum PEDF was not associated with renal decline over 3.1 years (37). Differences in the baseline patient characteristics, being all male and slightly older in the Veterans Affairs Diabetes Trial subjects, and study design, with shorter follow-up duration and a different definition of CKD progression, may have contributed to the differing results compared with our findings.

The current study had several limitations. First, the number of incident macroalbuminuria cases was relatively small (3 of 94 albuminuria cases); therefore we could not evaluate the microalbuminuria and macroalbuminuria endpoints separately. Second, we did not have a comprehensive quantitative data on UAE for all the subjects with normoalbuminuria, defined as a urinary albumin-to-creatinine ratio ≤30 μg/mg creatinine, because some of our baseline UAE readings were below the sensitivity limits of our laboratory. Nonetheless, in the Cox regression analysis, we had included microalbuminuria/albuminuria as a categorical variable. Because this was a longitudinal observational study, the treatment protocols for the patients in our cohort were not controlled, and therefore, potential confounders during the observation period may add to some bias in the analyses. Another limitation is that we measured only plasma PEDF at a single time point. We also did not measure T1DM-related autoantibodies to exclude T1DM or latent autoimmune diabetes of adults. However, insulin therapy was initiated at a median of 11 years (IQR 7–15) after diagnosis in those that were on insulin at baseline, making T1DM or latent autoimmune diabetes of adults less likely in our cohort. Lastly, our study was conducted at a single diabetes specialty center. Therefore, our findings would require further external validation. Because PEDF or neutralizing antibody against RAGE is implicated in the inhibition of AGE-induced oxidative stress generation in mesangial cells (23), measuring the plasma soluble isoform of RAGE (sRAGE), which correlated positively with circulating AGE and negatively with GFR (38), may provide additional information on the interaction between PEDF and AGE in the pathogenesis of DN.

In conclusion, plasma PEDF predicted the deterioration in renal function and development of microalbuminuria/albuminuria, during a median follow-up of 4 years, in patients with T2DM. Plasma PEDF may have a potential role in the early identification of diabetic patients at increased risk of nephropathy progression.
